# Identifying potential measures of stress and disturbance during a captive to wild African elephant reintegration

**DOI:** 10.1371/journal.pone.0291293

**Published:** 2023-10-03

**Authors:** Yolanda Pretorius, Tamara Eggeling, Andre Ganswindt

**Affiliations:** 1 Elephant Reintegration Trust, Port Alfred, South Africa; 2 Mammal Research Institute, University of Pretoria, Hatfield, South Africa; 3 Endocrine Research Laboratory, Department of Anatomy and Physiology, University of Pretoria, Pretoria, South Africa; University of Mississippi, UNITED STATES

## Abstract

There is increasing evidence of compromised welfare for elephants managed in captivity. Should such facilities eventually close, more elephants will need to be rehabilitated and reintegrated into the wild. The goal of such reintegration would be to restore any physical or psychological aspects of the elephant that may have been compromised in captivity, followed by introduction into a free-roaming system where they can interact with other elephants. However, to achieve this goal, the reintegration methods implemented need to be assessed to ensure that welfare remains the priority. The objective of this study was to test whether parameters generally associated with stress and disturbance in African elephants, respond to changes in potentially stressful environmental conditions, assessed at multiple temporal scales ranging from minutes to months. The main changes in environmental conditions that were investigated included the different phases of reintegration of a group of elephants from captivity into the wild. Stress and disturbance related parameters used for comparisons included physiological responses, namely the extent of temporal gland secretions (eTGS) and faecal glucocorticoid metabolite (fGCM) levels as well as behavioural responses, namely the display of stereotype and stress-related behaviours. Results showed that eTGS significantly increased during the initial release of the elephants compared to when in captivity. Stereotypic behaviours were only recorded during the captive phase and immediately ceased after release. Faecal GCM levels spiked in the first year after release before decreasing back to pre-reintegration levels during the third year. These findings indicate that fGCM levels, the eTGS and disturbance related behaviours all proved effective in explaining the changes in stress and disturbance experienced by elephants during the initial years after being reintegrated from captivity into the wild.

## Introduction

One of the biggest challenges facing conservation and wildlife managers today is to determine whether a particular management action is disturbing or causing stress for the species being managed that might compromise the animal’s well-being (often termed interchangeably as “welfare”). This is especially true for charismatic species, such as elephant, with high viewing value that are known to be socially complex and intelligent [1, 2]. Moreover, it is presumed that the well-being of animals will be better when they can roam freely compared to when they are kept in captivity. However, little scientific evidence exists to support this assumption, especially in the case of elephants. By definition, well-being refers to the concept of satisfying an animal’s basic behavioural and social requirements in an environment that causes minimal disturbance, stress and distress [3]. While welfare is defined similarly, Fraser [4] suggests well-being as the state of the animal whereas welfare covers the broader concept of social and ethical issues [5]. As both terms still cover concepts of addressing an animal’s needs appropriately, in this paper we have chosen to use well-being and welfare interchangeably.

An extension of evaluating the well-being of an animal is also to determine how they are able to cope with disturbance and stress (allostasis) [6]. A high level of well-being would mean an animal was effectively coping and adapting to internal needs and environmental demands by appropriately using and conserving its bodily resources [7]. Stress is a biological response that disrupts an animal’s homeostasis, and the response of an animal will depend on factors such as health, genetics, age and previous life experiences [8]. In response to stressors, animals may elicit certain stress responses, but it is not to say they aren’t coping or that it produces negative consequences. Stress becomes distress however when a stressor is so severe that an animal is no longer able to maintain homeostasis, resulting in redirection of biological resources such as energy to, for example, alter behaviour, instead of to other functions such as immune competence [9, 10]. It is at this point that well-being becomes compromised and it is this relationship that we are aiming to understand better through studies such as this.

Historically it was believed that once an elephant had been trained or kept captive it could no longer be reintroduced back into the wild (Brett Mitchell (Elephant Reintegration Trust), pers comm). Although there are cases of successful reintroduction of orphaned Asian elephants back into the wild [11–13], unfortunately little post release monitoring has been done so far [12]. Even fewer attempts of reintroducing adult Asian elephants have been undertaken when compared to African elephants [14]. In the last decade, more experience has been gained in southern Africa where two captive adult African elephants (*Loxodonta africana*) were successfully reintegrated to Pilanesberg National Park [15], four elephants to Gondwana Game Reserve, seven elephants to Khamab Kalahari Reserve, one elephant to Thula Thula Private Game Reserve, and seven elephants to Abu Camp, Botswana (Brett Mitchell (Elephant Reintegration Trust), pers comm). However, the behaviour of the elephants once released seems to vary according to the reintegration process. Other than the study on the release of three captive-raised young adult bulls in Botswana [16], there is still a lack of accessible scientific evidence surrounding reintroduction, the effects on elephant welfare, social situation and physiology; and the long-term behavioural effects on reintroduced captive elephants.

Determining whether the welfare of an elephant has been affected through environmental changes requires detailed observations over time to see how the presentation and frequency of certain behaviours may differ, from expected baselines, in order to make conclusions about an individual’s well-being. Although there is still much ground to be covered with regards to understanding the intricacies of elephant behaviour, certain pronounced behaviours have been found to reflect heightened stress more reliably. Stress in an elephant is defined as an increase in adrenal activity and/or a display of behaviours commonly accepted to be stress induced by animal behaviouralists, including, disturbance, distress, ambivalence or discomfort which can also manifest as stereotypic behaviours in captive animals [17–19].

Many studies on African elephants have also looked at physiological parameters as a means to more conclusively quantify the level of stress an elephant is experiencing, including examining glucocorticoid metabolite levels in the dung (fGCM) as well as the presence and extent of secretions from the temporal gland situated between the eye and the ear of the elephant (eTGS), which is associated with chemical communication and advertisement of the reproductive and/or emotional state of the animal [17]. Management actions such as translocation of elephants [20] as well as exposure to humans in the form of tourism [21] have all been shown to increase fGCM levels. However, studies on the effect of environmental disturbances, (e.g., season, reserve size and human presence) on eTGS in African elephants are rare, possibly because of large variation in the frequency and extent of secretions between bulls and cows and elephants of different ages [22].

The process of reintegrating a herd of elephants from captivity into the wild constitute of multiple steps that facilitate the transition from being fully dependent on people, with limited freedom of choice, to a point of being self-sufficient and free roaming. These stages would therefore represent significant changes to the conditions these elephants had been accustomed to for the duration of their lives in captivity. Through this process, such transitions provide the unique opportunity to observe the effects these potential disturbances and stressors can have on specific behaviours in the elephants.

The objective of this study was to test whether a variety of parameters (physiological and behavioral) generally associated with stress and disturbance in elephants increase or decrease within a herd that are reintegrated from captivity into the wild. Classical physiological responses to stress, namely fGCM levels and eTGS, were measured to quantify this. To substantiate the potential evidence for improved welfare of animals reintegrated from captive to wild status, we also included behavioural responses, namely the display of stereotype and stress-related behaviours by individual elephants across temporal scales ranging from minutes to months.

Our prediction was that all disturbance and stress-related parameters included in the study will initially increase as the elephants are released and experience new conditions, before declining to levels on par with or even lower than what they were in captivity.

## Methods

All invasive activities with the elephants (e.g. fitting of GPS collars or treatment of wounds) were conducted by the reserve management team where the study was done and the researchers were only involved in observing the elephants in the presence of the elephant handlers. The reserve had all dung samples analysed in a laboratory as part of their standard elephant monitoring protocol and granted permission to the researchers to use this data for inclusion in this paper. Approval was obtained from the reserve managers and elephant owners (Shambala Game Reserve Reg No 2000/021200/07 and Elephant Reintegration Trust IT1072017E) granting the authors (researchers) permission to observe the elephants throughout the reintegration process and providing ethical clearance for all data collection techniques used in the study.

### Study site

Shambala Private Game Reserve (SPGR) is a privately owned 10 000 ha reserve in the Waterberg region of South Africa (Limpopo Province). The reserve hosts a variety of large herbivore species such as impala (*Aepyceros melampus*), blue wildebeest (*Connochaetes taurinus*), zebra (*Equus quagga*) and giraffe (*Giraffa camelopardalis giraffe*), predators such as spotted hyenas (*Crocuta crocuta*), lion (*Panthera leo*) and leopard (*Panthera pardus*), as well as larger species like white rhinoceros (*Ceratotherium simum*), African buffalo (*Syncerus caffer*) and African elephant (*Loxodonta africana*). However, all the elephants on the reserve were historically kept in captivity for elephant-back safaris and only released to roam free on the reserve during the course of this study.

SPGR falls within the Waterberg mountain bushveld of the central bushveld bioregion of the savanna biome. This type of bushveld is dominated by rugged mountains with vegetation ranging from *Faurea saligna-Protea caffra* bushveld on higher slopes through to broadleaved deciduous bushveld on rocky mid- and footslopes. *Burkea africana-Terminalia sericea* savannas are typically found in the lower-lying valleys and on deeper sandy soils with a moderate to well-developed grass layer [23].

During the study, rainfall at the study site was as follows: 481 mm during the 2015/2016 season, 552 mm for the 2016/2017 season (15% higher than first season), 332 mm for the 2017/2018 season (31% lower than first season) and 363 mm for the 2018/2019 season (25% lower than first season).

### Study animals

Shambala Private Game Reserve acquired six captive African elephants (four bulls and two cows) in 2002 from a captive elephant facility in Zimbabwe. These elephants were originally captured as orphans after culling programs took place in the 1980’s in Gonarezhou National Park.

The Elephant Back Safari operation on SPGR was commercialized from 2004–2016. However, the Elephant Back Safaris only operated once a day for about an hour in the morning. The rest of the day the handlers took the elephants out onto the reserve to forage and in the evenings herded them back to their stables (a secured holding enclosure designed to keep animals separate from each other for captive purposes) where they were locked in for the night.

Between 2002 and 2015 the Reserve welcomed 6 calves into the herd, born to the original two cows and to one of the daughters of one of the original cows (Table 1). Three bulls were translocated to other reserves, one bull to KwaZulu-Natal in 2010 and two bulls to a reserve in the Waterberg in 2014.

**Table 1:** Demography of the African elephant population at the time of reintegration.

Elephant	Sex	Sire	Dam	Date of Birth	Age Group
Mickey	Male	Unknown	Unknown	1981	Adult
Anna	Female	Unknown	Unknown	1984	Adult
Mouse	Female	Unknown	Unknown	1985	Adult
Shambala	Female	Jumbo	Mouse	2002	Sub-adult
Madiba	Female	Jumbo	Anna	2003	Sub-adult
Dimpho	Female	Mickey	Mouse	2008	Juvenile
Kidibone	Female	Mickey	Anna	2009	Juvenile
Sabuka	Male	Gobisa	Mouse	2011	Juvenile
Temba	Male	Mickey	Shambala	2013	Suckling calf
Moya	Male	Mickey	Mouse	2014	Suckling calf
Unnamed	Male	Mickey	Shambala	2016	Died four days after birth
Shanduwa	Female	Mickey	Mouse	2017	Not included in study
Ndivhuwo	Female	Mickey	Shambala	2019	Not included in study

### Data collection

The reintegration process for the study animals was split into the following different stages: ridden, stables, boma, release, and year 1, 2 and 3 after release. Each of these stages represent different potentials for impact on welfare. The occurrence of all stress-related parameters observed in the study animals were compared during these different key stages of the reintegration including fGCM, eTGS, stereotype and stress-related behaviours. This was done at four different temporal scales which focused on: a) captivity status, b) reintegration phase, c) extreme infrequent disturbance events and d) common immediate disturbances.

#### Captivity status (monthly scale)

Comparisons were made between the two main periods of the study, namely the period during which the elephants were still kept in captivity (15 January to 20 May 2016) and the subsequent period when they were set free (21 May to 28 July 2016).

#### Reintegration phase (weekly scale)

Each of the two main periods were sub-divided into distinct phases as follows (Table 2):

- Phase 1 –Ridden (elephants still ridden by tourists as part of an elephant-back safari operation),
- Phase 2 –Stables (elephants not ridden but still kept in individual stables at night and herded by human handlers during the day)
- Phase 3 –Boma (elephants moved to an electrically fenced 17000 m^2^, open-air enclosure where they were closed in together at night, with food provided, while allowed to roam free within a 5 km radius of the boma during the day)
- Phase 4 –Release (gate of boma left open and elephants allowed to roam free on the reserve)
- Phase 5 –Post-release (elephants roaming the reserve after 5 weeks of the release. Although direct observations of the elephant by the researchers ceased on the 28^th^ of July, the field technicians employed by the reserve and who assisted the researchers in the field, continued to collect dung samples from all the individual elephants periodically for three years between 29 July 2016 to 29 July 2019. These samples were also included in the analysis of the dung data to see what the long-term trends were.

**Table 2:** Phases of reintegration of a group of African elephants with each phase’s prevailing environmental conditions.

Phase Nr	Phase Name	Duration	Elephant rides by tourist	Stabled at night	In boma at night	Access to boma	Herded during day	Herded to boma/stable at dusk
1	Ridden	15Jan-4Mar16	Yes	Yes	No	No	Yes	Yes
2	Stables	5-16Mar16	No	Yes	No	No	Yes	Yes
3	Boma	17Mar-20May16	No	No	Yes	Yes	No	Yes
4	Release	21May-27Jun16	No	No	No	Yes	No	No
5	Post-release	28Jun-28Jul16	No	No	No	No	No	No

#### Extreme infrequent disturbance events (daily scale)

Throughout the five reintegration phases of the study, note was taken of days during which the elephants were exposed to potential extreme disturbance events such as the fitting of tracking collars, physical treatment of wounds, injection of the bull with Gonadotropin Releasing Hormone (GnRH) (standard management procedure on the reserve to suppress testosterone and related aggression), aggressive physical interactions with other species such as rhinoceros and subsequent intervention by handlers and the initial movement of the elephant from the stables to the new enclosure, which would serve as their sleeping area. For the analysis, all extreme disturbance events were pooled together and their occurrence each day simply recorded as present or absent.

#### Common immediate disturbances (minute scale)

Throughout the five reintegration phases of the study, data was recorded on the occurrence of stress-related parameters during daily observation sessions. An observation session was defined as the period during which a fixed number of elephants were visible and environmental conditions such as the prevailing weather conditions, human presence and other natural disturbances did not change throughout the session. During each session the following variables were recorded: (i) session number, (ii) start and end time of session, (iii) general location of elephant on the reserve, (iv) estimated distance of the observer from the elephants and, (v) the number of elephants visible. Along with this information for each session, any immediate disturbances present in the environments were recorded as: (i) number of vehicles visible within 100 m of the elephant, (ii) other visible or audible human disturbances in the area of the elephant (elephant handlers or humans on foot closer than 100 m from elephant/loud sounds from lodge/sound of aircraft/loud sound of machinery), (iii) natural disturbances (fire/ thunderstorm/ other large mammal species within 50 m of the elephant).

For the analysis, all common immediate disturbances were pooled together and their occurrence during an observation session simply recorded as present or absent.

### Physiological stress parameters

#### Faecal glucocorticoid metabolites

A sum of 362 faecal samples (Table 3) were collected on 154 days in total over the study period. Samples were only collected within one hour of an elephant seen defecating if there was an opportunity to retrieve it. Each sample that was collected from the fresh faecal material was approximately 25 gram (golf ball size) and was collected from the middle of at least two boli, which was then mixed and stored in a plastic Ziploc bag. To limit fGCM alteration post-defecation, the samples were put in a cooler box with ice packs whilst in the field and transferred to a freezer within 4 hours, following Webber et al. [24]. Samples were kept frozen until analysis of their fGCM levels at the Endocrinology Laboratory of the University of Pretoria. Faecal samples were lyophilized, pulverized and sifted using a nylon mesh strainer to remove fibrous material as described by Fiess et al. [25]. Between 0.050–0.055 g of the faecal powder was then extracted with 80% ethanol in water (3 ml). The suspensions were vortexed for 15 min and subsequently centrifuged at 1500 *g* for 10 min. The supernatants formed were transferred into microcentrifuge tubes and stored at −20°C for further analysis.

**Table 3:** Table summarising the number of dung samples collected and analysed per individual at each stage of the study (n = 362).

	Ridden	Stables	Boma	Release	Post-release	Year 1	Year 2	Year 3	Total
Mickey	9	5	17	12	8	20			71
Anna	9	5	9	6	8	10	2	6	55
Mouse	8	5	10	6	6	5	2	3	45
Madiba	8	5	9	7	7	5	2	4	47
Shambala	8	5	8	9	6	5	1	2	44
Dimpho	6	4	7	5	6	2	3	2	35
Kidibone	6	4	7	5	5	3	4	3	37
Moya	5	2		3	3			2	15
Themba	5	2		3	1			2	13
Total	64	37	67	56	50	50	14	24	362

The resulting extracts were measured for immunoreactive fGCM concentrations using an 11-oxoetiocholanolone enzyme immunoassay (EIA), detecting fGCMs with a 5*β*-3*α*-ol-11-one structure. This EIA has been shown to reliably detect adrenocortical function in African elephants [26, 27]. Detailed assay characteristics, including full descriptions of the assay components and cross-reactivities, have been provided by Möstl et al. [28]. Sensitivity (at 90% binding) of the assay was 1.5 ng/g dry weight (DW). Intra- and Inter-assay coefficients of variance determined by repeated measurements of high and low value quality controls were 4.87% and 6.60% as well as 15.60% and 16.58%, respectively. Serial dilutions of faecal extracts gave displacement curves that were parallel to the respective standard curves for both assays with a relative variation of the slope of the trend lines *<* 3%. All steroid concentrations are expressed per mass of faecal DW matter.

Because of retention time in the gastro-intestinal tract, it is difficult to ascribe an elevation in fGCM levels to a specific event. Retention times in African elephants vary significantly, from 7 [29] to 30 hours [30, 31] depending on factors such as the diet of the animal. Therefore, the faecal data in this study were only used for comparisons between the captivity status of the elephant and the different phases of reintegration, as these could be pooled across weeks and months.

#### Extent of temporal gland secretions

To measure temporal gland secretions, data was collected whilst observing the elephant herd for 86 days, which comprised of 578 observation sessions.

At the start of every new observation session, all the visible elephants were scanned using binoculars to check for the presence of fresh temporal gland secretions. If an elephant did not have any fresh secretions, the number 0 was recorded below the elephant’s name. If fresh secretions were present, the extent was indicated below the relevant elephant’s name as follows: 1 –a spot around the opening of the temporal gland, 2 –a stripe halfway down the face, 3 –a stripe all the way down the face, 4 –a thick stream of secretion all the way down the face to the chin of the elephant. Temporal gland secretion in elephants is known to be an instantaneous response to an environmental stimulus [1, 32]. However, to eliminate the potential for pseudo-replication, all sessions less than 20 minutes were removed from the dataset before analysis.

### Behavioural stress parameters

#### Disturbance related behaviours

The following behaviours generally associated with disturbance, distress, ambivalence, or discomfort were grouped together as disturbance related behaviours [19, 33, 34] for the purpose of this study. These behaviours included: walking warily with tail up, head held high and ears out, fleeing silently with ears flat against body and tail up (where fleeing is the action of quickly moving away from an object or disturbance), fleeing with ears out, tail up and vocalizing, head shaking, sudden pause whilst listening with head up and ears out, bundling together in a defensive fashion with heads facing out, twitching and twirling of trunk as well as vocalizations such as crying (a short sound produced by an infant or calf under the age of five years in some form of mild distress) and roaring (a highly variable roaring, bellowing, screaming, shrieking, or squealing call typically lasting 1–2 seconds in duration) [19].

Data on disturbance related behaviours were collected in two ways. First, the occurrence of the behaviours mentioned above were measured during 578 observation sessions, whilst observing all elephants visible or audible at a sighting. At least 65 observation sessions were included for every phase of reintegration in the analysis. For analysis, the number of disturbance related behaviours per session were calculated as a frequency (behaviour frequency per minute).

Second, detailed focal observations were made of each individual elephant. This was done using 10-minute video recordings. At the start of each focal observation the elephant’s name was recorded along with the start time of the focal and the observation session number associated with it. After this, the main activity the elephant was involved in was recorded (feeding/ resting/ drinking/ self-care/ moving/ environmental interaction/ social interaction/ other) and every time this activity changed or one of the stress-related behaviours occurred, the current time and behaviour was recorded. This allowed for the duration of each activity and behaviour to be calculated, which further enabled calculation of a frequency (behaviour duration per focal observation interval) that could be used in the analysis.

#### Stereotype behaviours

Along with stress-related behaviours recorded during observation sessions of the entire group of elephants, note was also taken of the occurrence of stereotype behaviours, including head bobbing (consistently bouncing head up and down), and rocking or swaying body from side to side (at a consistent pace) [35]. As these behaviours are generally only associated with captivity and expected to be sporadic, if present at all, only comparisons associated with the captivity status and phase of reintegration were considered in the analysis.

### Data analysis

According to the Konglomorov-Smirnov Test the fGCM concentration, eTGS, and behavioural data sets were not normally distributed. Hence, non-parametric tests were used for analysis and all the data collected on individual elephants for each of the parameters were averaged to allow for pairwise comparisons between the different periods. For paired comparisons between only two periods such as for the captivity status (captive/free), the presence or absence of extreme disturbance events and/or common immediate disturbances, a Wilcoxon Signed Rank test was used. For paired comparisons between more than two groups such as between the different phases of reintegration for the temporal gland secretion dataset, the Friedman repeated measures analysis of variance on ranks was used. Where there were missing values for some individual elephants in the focal disturbance and dung data sets, the Skillings-Mack Test was used to do a pairwise comparison between the different phases of reintegration. For group observations of disturbance related and stereotype behaviours as well as for differences between functional sex and age groups, the Mann-Whitney U test was used for comparisons between only two periods and the Kruskal Wallis test for comparisons between more than two periods. To test for differences between individual elephants for the different parameters the Kruskal Wallis test was used (Table 4).

**Table 4:** A summary of the statistical tests used in the analysis of data for a group of African elephant and for individuals within the group.

	Temporal scale:	MONTHLY	WEEKLY	DAILY	MINUTE	
	Independent variable:	*Captivity status*	*Reintegration phase*	*Extreme infrequent disturbance events*	*Common immediate disturbances*	*Individual Elephant*
**Dependent variable:**	**Classes:**	*Captive/ Free*	*Ridden/ Stables/ Boma/ Release/ Post-release*	*Present/ Absent*	*Present/ Absent*	*10 identifiable elephants*
***Feacal Glucocorticoid metabolites***	*measured in ug/g DW*	Wilcoxon Signed Rank Test	Skillings-Mack Test	-	-	Kruskal-Wallis Test
***Extent of Temporal Gland Secretion***	*1/ 2/ 3/ 4*	Wilcoxon Signed Rank Test	Friedman repeated measures	Wilcoxon Signed Rank Test	Wilcoxon Signed Rank Test	Kruskal-Wallis Test
***Disturbance related behaviours***	*Frequency*	Mann-Whitney U test (Wilcoxon Signed Rank Test)	Kruskal Wallis Test (Skillings-Mack Test)	Mann-Whitney U test (Wilcoxon Signed Rank Test)	Mann-Whitney U test (Wilcoxon Signed Rank Test)	-
***Stereotype behaviours***	*Present/ Absent*	Mann-Whitney U test	-			

*tests in brackets are for the analysis of data on individual elephants

To control for the probability of committing a type I error the Bonferroni correction was applied to all post hoc tests including the Tukey and Dunn’s test for differences between groups.

## Results

### Captivity status

**Fig 1:**
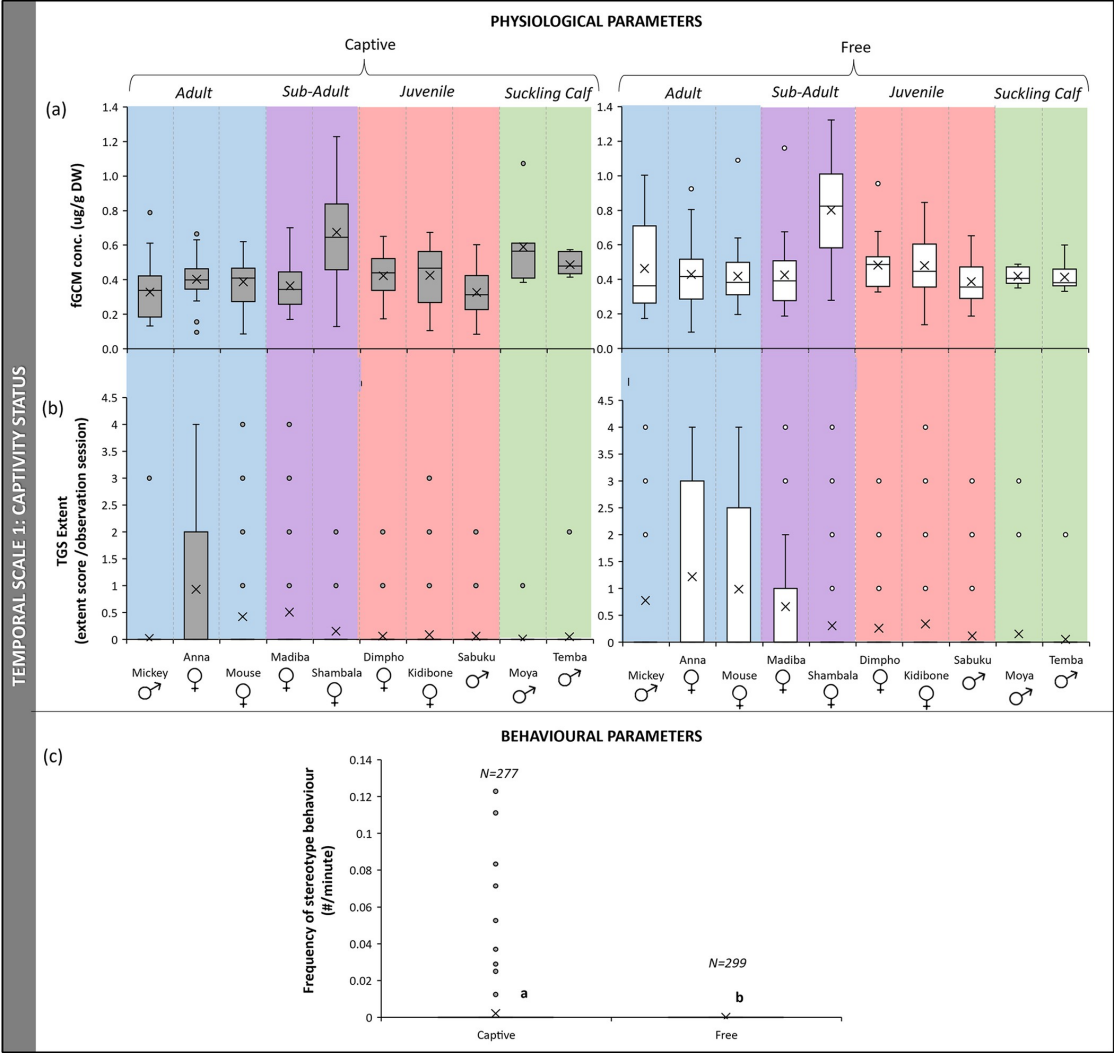
Boxplots showing changes in (a) fGCM concentration and (b) extent of temporal gland secretion for each individual elephant (coloured sections indicate age class of elephant) and, (c) frequency of stereotypic behaviours (expressed as number per minute) of a group of African elephant, before and after release from captivity into the wild (different letters on bars in graphs indicate significance, where the same letter is indicated there is no significant difference and p > 0.05; “X” marks the mean).

There was no difference between fGCM levels before and three months after release (N = 10, z-score = 1.07, p > 0.05), but the extent of temporal gland secretion was significantly higher after release compared to captivity (N = 10, z-score = 2.803, p<0.05) (Fig 1). There was no significant difference between the frequency of disturbance related behaviours measured during focal (N = 10, z-score = 0.968, p>0.05) and group (N = 576, U = 65654, p>0.05) observations between captivity and being free (Fig 1). No stereotypic behaviours were observed after the elephants were released (N = 576, U = 37091, p<0.05) (Fig 1).

### Reintegration phase

**Fig 2:**
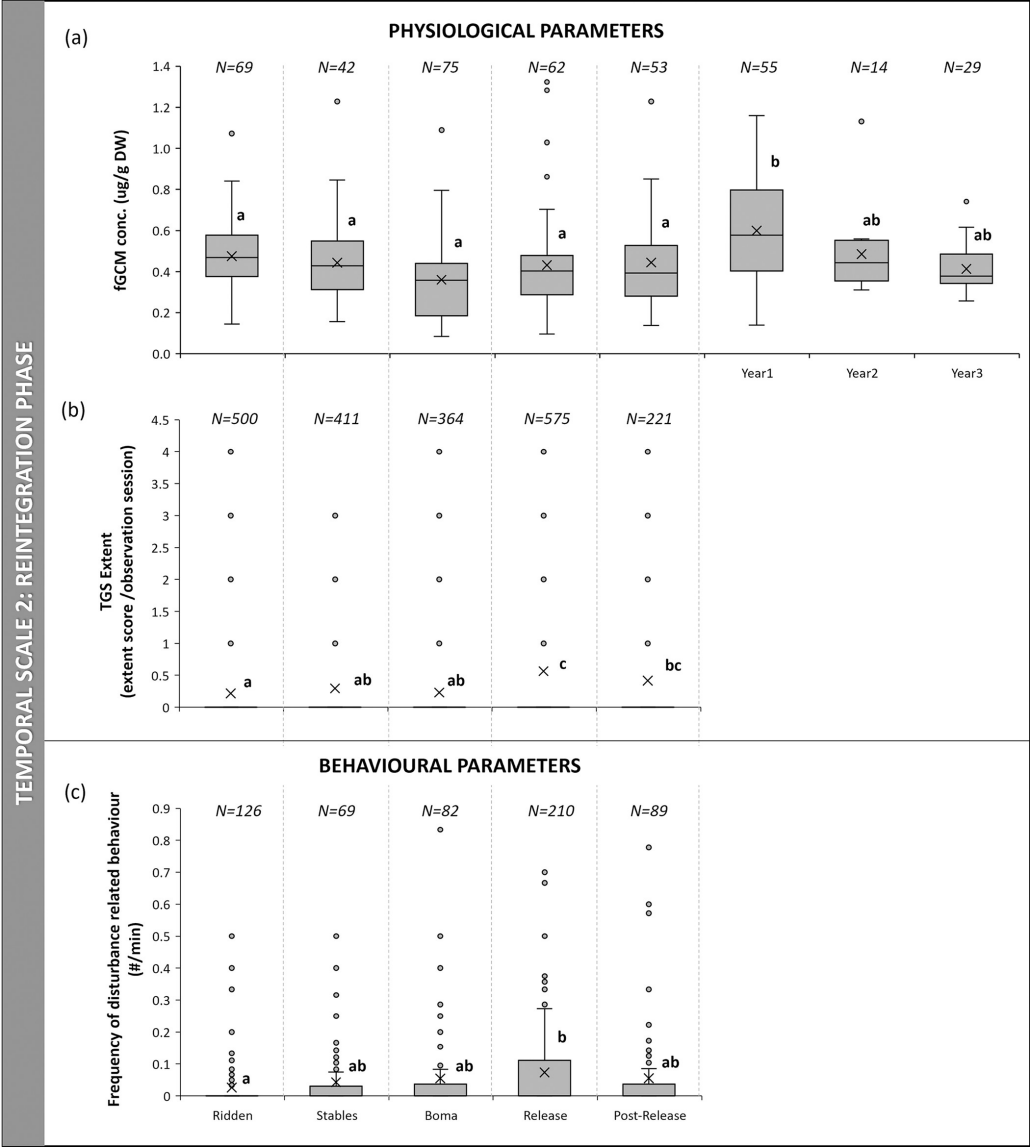
Boxplots of stress response of a group of African elephants during different phases of reintegration into the wild: (a) fGCM concentrations, (b) extent of temporal gland secretion and, (c) frequency of disturbance-related behaviours for group observations (number per minute) (different letters on bars indicate significance, where the same letter is indicated there is no significant difference and p > 0.05; “X” marks the mean).

Faecal GCM levels differed significantly among the different phases of reintegration (N = 10, Q (critical value) = 14.07, Q (observed value) = 24.72, p < 0.05), with the first year after the release having significantly higher levels than the preceding phases. The third year was significantly lower than the first year before the Bonferroni correction (p = 0.004) but not thereafter. Prior to the Bonferroni correction, fGCM was significantly lower when elephants were kept in the boma compared to the stable (p = 0.024), the ridden (p = 0.019), the post-release (p = 0.005) and the second year (p = 0.01) phases (Fig 2).

The eTGS also differed among reintegration phases (N = 10, Q = 15.76, p < 0.05) with eTGS extent being significantly higher during the release phase compared to the boma and ridden phases (also higher than stable phase before the Bonferroni correction (p = 0.041)). Elephants also secreted significantly more during the adapt phase compared to the ridden phase (Fig 2).

Disturbance-related behaviours did not differ significantly between phases during focal observations (N = 10, Q (critical value) = 9.49, Q (observed value) = 2.73, p > 0.05). However, disturbance-related behaviours were different between phases during group observation sessions (N = 576, H = 25, p < 0.05) with the release phase having significantly higher frequencies than the ridden phase, and prior to the Bonferroni correction, also than the boma (p = 0.025) and adapt (p = 0.011) phases (Fig 2). Stereotypic behaviours only occurred during the ridden and stable phase and not during any of the other successive phases.

### Extreme infrequent disturbance events

**Fig 3:**
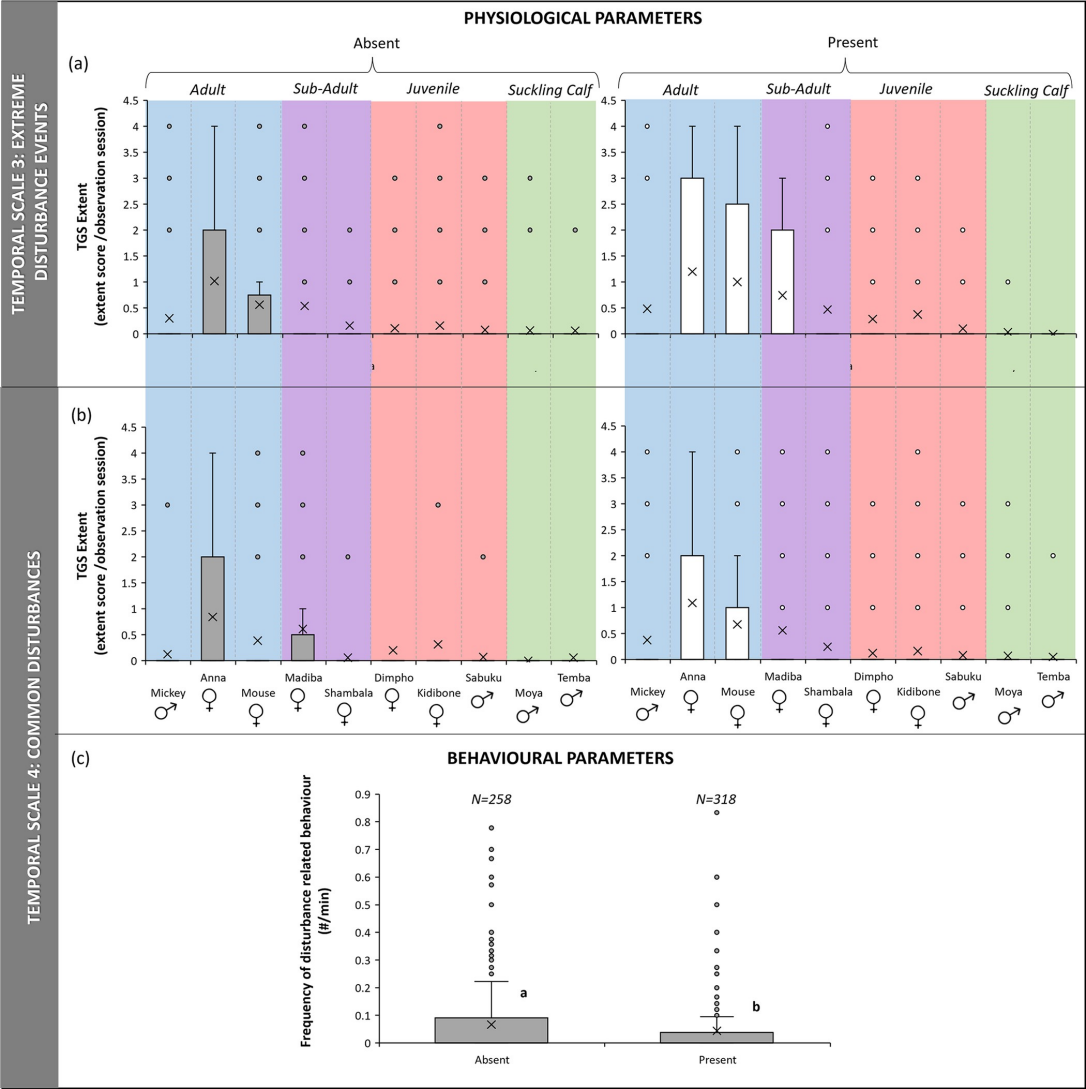
Stress response of a group of African elephants to the presence of extreme infrequent (a) and common immediate disturbances (b and c) in their environment. (a) and (b) represent extent of temporal gland secretion for each individual elephant (coloured sections indicate age class of elephant) and, (c) the frequency of stress-related behaviour (expressed as number per minute) of the group of elephants (different letters on bars in graphs indicate significance, where the same letter is indicated there is no significant difference and p > 0.05; “X” marks the mean).

The eTGS was significantly higher for the elephant study group during days in which extreme disturbance events occurred (N = 10, z-score = 2,293, p < 0.05) (Fig 3). However, there was no difference in the frequency of disturbance-related behaviours for group observation sessions or during focal observations.

### Common immediate disturbances

The eTGS (N = 10, z-score = 1.478, p > 0.05) and disturbance-related behaviours for focal observations (N = 10, z-score = 0.051, p > 0.05) were not significantly different for the elephants during sessions when common disturbances were present in the immediate environment compared to when not. In contrast, disturbance-related behaviours during group observations (N = 576, U = 17377, p < 0.05) were significantly lower in the presence of common disturbances (Fig 3).

Overall, there were significant differences between individual elephants for fGCM levels (N = 399, H = 72.27, p < 0.0.5) and the eTGS (N = 2071, H = 89.7, p < 0.05). The sub-adult cow, Shambala, had significantly higher fGCM levels compared to most of the other elephants, whereas the adult cow, Anna, had significantly higher eTGS recorded.

## Discussion

With increasing consideration being given towards animal welfare, it becomes critical to ensure that all interventions employed to this end are effective in improving animal well-being. Interventions risk creating unnecessary disturbances, so even if well-intentioned, situations could be worsened if not sufficiently researched. This study was aimed at measuring whether reintegration of African elephants from captivity into the wild, changed the occurrence of parameters generally associated with stress and disturbance in elephants, including the extent of temporal gland secretions (eTGS), faecal glucocorticoid metabolite (fGCM) levels and the display of stereotype and disturbance-related behaviours. At broader temporal scales, the eTGS increased when elephants were released from captivity. Whereas stereotypic behaviours completely ceased after release.

When investigating these changes at a finer temporal resolution by comparing the different phases of reintegration, the reason for the broader scale changes becomes clearer and fits the prediction that stress increases after the initial release, but then declines again.

In accordance with the findings of Kosaruk et al. [36], fGCM concentrations were not higher when elephants were still used for elephant back safaris compared to when the safaris ceased and elephants were only stabled, whereas Grotto et al. [37] found that elephants that participated in elephant-back-safari activities showed significant decreases in fGCM concentrations when elephant-back-safaris were discontinued. An alternative explanation for our findings could be that there is a physiological delay post-release. Although not conclusive, support for a lag response in fGCM levels can be substantiated when comparing the level of response from the different parameters investigated in this study during the initial release phase. Both temporal gland secretions and disturbance related behaviours are instantaneous responses and were significantly higher during the initial release. However, fGCM levels were only significantly higher later in the first year after release. One study conducted in Kruger National Park by Ganswindt et al. [27] showed a similarly interesting lag in increased fGCM levels following an injury in an African elephant bull. Although the fGCM levels did increase slightly above the individual’s baseline from the time the injury was first recorded, the threshold for elevated fGCM levels was only actually exceeded a month into the three-month injury period where it remained elevated for several weeks before the injury healed and stopped causing discomfort.

Even though the eTGS and disturbance related behaviours could not be measured more than three months after release, the decline in fGCM levels during the third year compared to the significant rise of levels during the first year post-release to levels similar to pre-release seem to indicate that the reintegration did not compromise their overall welfare in terms of physiological stress. This is true despite a 31% lower rainfall in year three than in the first year of release. Rainfall is causally related to vegetation biomass and therefore available food [38]. The elephants did not receive the food supplements after release which they were given in captivity up until the boma phase. This should have resulted in increased nutritional stress, especially in year three, as found by many other studies conducted on changes in seasonal fGCM levels in African elephants [27, 35, 39]. Additionally, despite the potential increased nutritional stress after release, the elephants never returned to the boma, which they would have associated with easy access to highly palatable and nutritious food supplements.

Although the eTGS was significantly higher on days when elephants were exposed to extreme disturbance events as well as during sessions when more common disturbances were present, disturbance related behaviours during group observations in the presence of common disturbances showed the opposite trend. This result emphasizes the complexity of interpreting the behaviour of animals that have been exposed and trained by humans in captivity. Most of the environmental factors classified as disturbances in this study were about the presence and proximity of humans and human activity around the elephants. In wild African elephants, increased activity and proximity of humans (e.g., tourists) have been shown to increase vigilance, avoidance and aggressive behaviour [40]. However, the elephants in the current study were all raised in close proximity to humans and were exposed to human activity on a daily basis. Hence, the absence of humans close by could lead to insecurity and uncertainty which would explain why behaviours associated with discomfort, ambivalence, frustration and anxiety, measured as a proxy for stress in this study, were significantly higher when humans were not close by. This also explains why these disturbance related behaviours increased significantly for group observations during the initial release phase as humans were suddenly keeping their distance and not controlling the elephant’s behaviour. After the first five weeks of release the occurrence of these behaviours declined.

It might seem contradictory that the eTGS increased both during the release phase as well as in the close proximity of humans and human activity. However, temporal gland secretions does not only occur when an elephant is experiencing stress but also when an elephant is very excited, such as when a family member who has been absent from the herd for a while joins up again [1]. Therefore, the eTGS needs to be considered within its context and alongside other parameters to correctly interpret.

The lack of change in disturbance related behaviours measured during focal observations between the different reintegration periods and temporal scales might be because of small sample sizes that did not provide sufficient statistical rigor to deal with the variation in disturbance related behaviours among individual elephants.

## Conclusion

This study aimed to test whether a variety of parameters generally associated with stress in elephants, increase or decrease within a herd that are reintegrated from captivity into the wild. Results indicate that fGCM levels, eTGS and disturbance related behaviours from group observations all proved effective in explaining the changes in stress experienced by elephants when they are reintegrated from captivity into the wild. However, some of these parameters seem to be better indicators at specific temporal scales and therefore further studies with larger sample sizes and comparisons across different populations is required.

Moreover, the study serves as an example of how African elephants that have been in captivity for more than 20 years can be successfully reintegrated without compromising the welfare of the animals in the context of stress. Not only did our findings illustrate this, but the successful birth of two calves to this herd during the study period also acts as a good indicator of fitness.

With the growing awareness among tourists and organisations [41] towards the negative effects of captivity on elephants and the negative publicity around these, such findings indicate that there are solutions for when elephants from captive backgrounds need to be released into the wild.

## Supporting information

S1 Data (XLSX)
